# Factors associated with unmet need for contraception in Mexico: evidence from the National Survey of Demographic Dynamics 2014

**DOI:** 10.1186/s12889-018-5439-0

**Published:** 2018-04-24

**Authors:** Fatima Juarez, Cecilia Gayet, Gabriela Mejia-Pailles

**Affiliations:** 1 El Colegio de Mexico, Mexico City, Mexico; 2 Facultad Latinoamericana de Ciencias Sociales Mexico, Mexico City, Mexico; 3 Independent Consultant, Mexico City, Mexico

**Keywords:** Unmet need for contraception, Mexico, Contraception among married and single women

## Abstract

**Background:**

Worldwide, the importance of contraception to control fertility has been recognized. A useful indicator of the gap between reproductive preferences and the provision of contraception is “unmet need for contraception”. The aims of this paper are to estimate the levels of unmet need for contraception among married and single women, and to explore factors associated with unmet need for contraception for spacing and limiting births in Mexico.

**Methods:**

We used the Mexican National Survey of Demographic Dynamics 2014, using a sub-sample of 56,797 sexually active women aged 15–49 years who were either currently in union or who had never been in union to estimate the prevalence of unmet need for spacing and limiting births. We applied multivariable binary logistic regressions to examine the relationship between unmet need for spacing and limiting considering associated factors.

**Results:**

Unmet need for contraception was estimated at 11.5% among women in union (6.4% limiting; 5.1% spacing), and 28.9% for women never in union (8% limiting; 20.9% spacing). In the logistic regression for unmet need for spacing, the likelihood was statistically significant associated with younger women (OR = 6.8; CI = 2.95–15.48); women never in union (OR = 1.6; CI = 1.40–1.79); low levels of education (OR = 1.4; CI = 1.26–1.56); and residing in poor regions (OR = 1.9; CI = 1.52–2.49). Those with full access to public services were significantly less likely to have unmet need for spacing (OR = 0.8; CI = 0.66–0.88).

In the logistic regression for unmet need for limiting, being younger (OR = 6.3; CI = 4.73–8.27), never in union and sexually active (OR = 3.0; CI = 2.47–3.54); with less schooling (OR 1.13; CI: 1.02–1.26); rural residence (OR = 1.2; CI = 1.07–1.32); and residing in poor regions (OR = 1.5; CI = 1.23–1.93) were factors positively associated with this unmet need. Women with private health services were the least likely to have unmet need for limiting (OR = 0.5; CI = 0.37–0.77).

**Conclusions:**

Younger women currently in union and never in union had the highest unmet needs of contraception for spacing and limiting. The results from this study suggest that in Mexico family planning services must prioritize the contraception needs of all young women, both in union and not in union, with appropriate and suitable services to cover their needs.

## Background

Globally, the importance of using contraceptives to regulate fertility has been recognized [1]. This is a key aspect of the reproductive health of women who wish to space or limit childbearing. A useful measure of the gap between women’s reproductive desires and the provision of health services is the estimation of unmet need for contraception. This indicator refers to women who wish to space or limit births but who do not use contraceptive methods to achieve this [2–5]. This indicator is important not only because it informs and guides contraceptive services, but also because it shows how far a country has ensured compliance with the reproductive health rights of its population. In Latin America and the Caribbean, there was a large increase in contraceptive use between 1970 and 2010 and a decrease in unmet need for contraception [4]. As Bongaarts states [6], as countries move forward in the demographic transition, the level of unmet demand for contraception drops.

Mexico has witnessed a significant reduction in fertility rates since the mid-1970s, declining from 7 children per woman at that time to 2.21 children per woman in 2014 [7, 8]. This decline was largely due to the work of public health services that provided contraception in all regions of the country. The proportion of married or in-union women using contraception rose from 30% in the mid-1970s to 72.3% in 2014 [7]. These changes occurred in combination with other important social phenomena: increasing urbanisation, expansion of schooling -especially of women-, and greater participation of women in the labour market, which, together with the introduction of modern contraceptive methods, allowed women to limit their family size [9]. However, once this rapid fall in fertility had been observed, these contraceptive policies were reduced as it was believed that the decline in fertility and the increased use of contraception use would continue. Since 2000, however, progress made regarding contraceptive prevalence and unmet need for contraception has stalled. This is combined with recent changes in sexual and reproductive behaviours, observed in an earlier sexual debut among women, an increasing gap between sexual initiation and union, and an increase in the desire to limit childbearing at earlier ages.

Therefore, while there has been an increase in the proportion of women who wish to space and limit childbearing, a stagnation in contraceptive supply programmes has been observed. In addition, another unresolved issue related to the decline in fertility has been the persistence of differences between socioeconomic groups [10]. Hence, there is the need to investigate the level of unmet need for contraception in Mexico and also to identify the factors that further hinder the use of contraceptives to meet the reproductive desires of women.

The indicator of unmet need for contraception has been widely used in developing countries, especially in those countries that have participated in the Demographic and Health Surveys (DHS) programme. It is a complex measure, which has international comparisons, and whose algorithm has changed over time. Since 2012, a revised version has been used which has gained consensus [3, 4]. Mexico has conducted a similar survey to the DHS named the National Survey of Demographic Dynamics (Encuesta Nacional de la Dinámica Demográfica –ENADID- in Spanish). Unlike the DHS surveys, the ENADID did not initially include all the questions necessary for the construction of the indicator of ‘unmet need’. However, in 2014, for the first time, ENADID included all of the questions needed to estimate the indicator that could be compared internationally. In addition, as has been recently proposed, the indicator can be calculated not only for married women, but also for sexually active unmarried women [4, 11, 12], and with the information from ENADID 2014, it can be estimated for Mexico. Thus, the objectives of this article are to estimate the levels of unmet need for contraception among married women and never married women, and to explore the factors associated with unmet need for contraception to space or limit births.

## Methods

### Study design

Secondary data analysis was conducted. The data comes from a 2014 cross-sectional national demographic survey of Mexico that is a random sample with national, state level and urban-rural representativeness, collected by the National Institute of Statistics and Geography (Instituto Nacional de Estadística y Geografía –INEGI- in Spanish). The sample included 101,389 households. In each selected household, all women aged 15 to 54 were interviewed [13]. The completion rate was 87% (2.9% had incomplete interviews and 9.2% had no response) [13]. For this analysis, the observation unit is women of reproductive age from 15 to 49 years, in a union (formal or consensual) and ‘never in union’ women who had had sex in the 3 months prior to the survey, totalling 56,797 cases.

### Setting

Mexico is a middle-income country, located in Latin America, with over 119 million inhabitants according to the Intercensal Survey of 2015, and has an annual population growth rate of 1.4% [14]. The population is mostly urban, and only 21% is considered as rural, i.e. living in localities of fewer than 2500 inhabitants [15]. The country has a young population, with 9 people of working age for each person aged 65 and over [16]. In Mexico, the increase in schooling has been notable, but it still shows lags and serves as an indicator of social strata [17]. Considering the population of 15 years and older, women had on average 9 years of schooling and men 9.3 in 2015 [14]. In 1974, an article in the constitution was changed regarding fertility regulation. It established that couples had the right to decide how many children they wanted and when to have them. This legislation is still valid at the present time. Family planning services have been established through public health services.

Mexico offers a range of different access to health services, all supervised by the Ministry of Health, but with differences in the quality of service. Affiliation to the health system (denoted as Social Security of Health Services) is not determined by need, but by a person’s job. Workers employed in the formal labour market (those who are paid a salary and pay taxes to the government) are insured by the government through different types of health insurance, depending on whether the employee works for the private sector (IMSS) or public sector (ISSSTE, and other government health services for employees of the army, navy, etc.). Belonging to the Social Security System is compulsory for employees in the formal sector, where a percentage of the employee’s salary is deducted to pay for it. Employees also have the right to private health insurance paid out of their own pockets, which can be costly. The rest of the population who are not employed in a formal job (workers in the informal and agricultural sector, or unemployed) can receive services from the Ministry of Health (Secretaría de Salud –SSA- in Spanish) through the state health services and also from *IMSS- Prospera* (which serves the uninsured rural population) or the “Popular Insurance” (Seguro Popular –SP- in Spanish). The latter is a new public health insurance scheme for low income groups and those enrolled in it access health services through the SSA [16]. As for health services coverage, in 2013, Mexico was considered to be the country with the lowest service coverage of the OECD countries: bed density was 1.6 beds per thousand inhabitants, and there were 2.2 practising doctors and 2.6 practising nurses per 1000 population [16].

### Statistical analysis

The main variable is unmet need for contraception, which is divided into need for spacing and need for limiting births. For this estimation, it was also necessary to consider women who were using contraception to space or limit fertility and those who did not need to use contraceptives either because they want to have children or were infecund. This last group, made up of 11.5% of all women of reproductive age, was excluded from the statistical model.

We compared women who were currently using contraception for spacing with those women who were not using contraception but who wished to delay their next birth for at least two or more years (unmet need for spacing). We also compared women who were currently using contraception to limit their fertility with those women who did not want any more children but were not using contraception (unmet need for limiting). Hence, the first dependent variable was “unmet need for spacing” with values of “0” for women who were using contraception to space and “1” for women who wanted to wait at least two years to have a child but were not using contraception. The second dependent variable was “unmet need for limiting”, with values of “0” for women who were using contraception to limit their fertility, and “1” for women who did not want more children and were not using contraception to limit their fertility. For our study, current users of contraception included those using both modern and traditional methods.

Associated factors in our study included 5-year age groups, marital status, urban-rural residence, education, region of residence, number of live births, and access to health services. Age was grouped in five years ranging from 15 to 19 years up to 45–49 years. We defined “women in union” as women in formal marriages and consensual unions. “Never in union women” consisted of women who, at the time of the survey, had never been married or in a consensual union. We excluded from the analysis women who had formerly been in a union due to the small numbers of cases. Sexually active women referred to women who had had sex in the 3 months prior to the date of the survey. Type of residence (urban or rural) used the official Mexican definition *– ‘*rural’ referring to localities with fewer than 2500 inhabitants. Education consisted of two categories: women with less than 10 years of schooling (basic compulsory education in Mexico) and women with 10 or more years of schooling. Mexico is not officially divided into regions so the 32 states were grouped according to the percentage of the population in poverty reported by The National Council for the Evaluation of Social Development Policy (Consejo Nacional de Evaluación de la Política de Desarrollo Social -CONEVAL-in Spanish) for the year 2014 [18] with the exception of Mexico City, which was considered as a region alone because it is very different to the rest of the country (lower fertility, more infrastructure, more health services, more educated population, and ranked as the region with the second lowest poverty rate by CONEVAL). The regions were therefore ranked as follows: Region 1: Mexico City; Region 2: States with less than 40% of population in poverty - Nuevo León, Baja California, Sonora, Coahuila, Baja California Sur, Querétaro, Colima, Chihuahua, Aguascalientes, Jalisco, Quintana Roo, Tamaulipas, Sinaloa; Region 3: states with between 40% and less than 65% of the population in poverty - Nayarit, Durango, Campeche, Yucatán, Guanajuato, San Luis Potosí, Tabasco, México, Morelos, Zacatecas, Hidalgo, Veracruz, Tlaxcala, Michoacán, Puebla and Region 4: with 65% or more of their population living in poverty - Guerrero, Oaxaca, Chiapas. The covariate of number of births was grouped as 0 children, 1, 2, and 3 or more children. We coded access to health services in four categories according to the quality of service from lowest to highest as follows: no social security health services (the worst scenario), partial access to public health services (workers in the informal sector covered by the public health services SSA and those enrolled in the Seguro Popular), full access to public health services (fully employed workers in the formal sector with access to IMSS/ISSSTE/or other similar services) and private health services.

STATA 13 (StataCorp., College Station, Texas, USA, 2013) was used for the analysis and all data was weighted. An analysis of the proportion of women with met need and unmet need for spacing and limiting considering marital status was conducted. Multivariable analysis was performed running binary logistic regressions to examine the relationship between “unmet need” (for spacing or for limiting) and independent variables. Separate models for each dependent variable were used. Before conducting the analyses, the multicollinearity between covariates was assessed using polychoric correlations, adjusting to re-scale marital status and social security into ordinal covariates. Highly correlated variables (number of live births) were removed from the models. Confidence intervals of 95% were calculated and *p* < 0.05 was considered statistically significant.

### Ethics

This study is based on secondary analysis of publicly available data; hence no ethical approval was required from our institutions. No permission is required to access and use these datasets; they are freely available.

## Results

The sample population consisted of 51,758 women in union and 5039 women never in union who were sexually active in the three months prior to the survey. Of the total number of women never in union interviewed (29,375), 17.2% reported having had sex in the three months prior to the survey, a subgroup to be considered in this study according to the definition of unmet need for contraception. To give evidence of the increasing proportion of women in need of contraception in recent years, we compared the percentage of women who had had sexual intercourse before age 20 for older and young cohorts. For the cohorts who were 40 to 44 years at the time of the survey, the proportion who had had sexual intercourse before the age of 20 was 52.9%. This percentage increased to 61.3% for the cohort aged 20 to 24 years at the time of the survey. This data is informative at aggregate level and is not included at the individual level in the model. The age distribution of the women in the study reflected the demographic composition of the country, with a mean age of 33.5 years (SD ± 8.9). Most of the women were urban (77%), with schooling less than 10 years (61%), and 15% had no health insurance.

Table 1 presents the overall estimates of unmet need for contraception by subgroup of population. The overall prevalence for unmet need for contraception was 11.5% for women currently in union (6.4% for limiting and 5.1% for spacing) and 28.9% for sexually active women never in union (8% for limiting and 20.9% for spacing). Among women currently in union, 57.4% were using contraception to limit and 15.2% to space. Among sexually active women never in union, 19.8% were using contraception to limit and 43.8% to space. The descriptive results showed, as expected, that younger women, both currently in union and never in union, use contraceptive methods more for spacing, and older women use contraception more for limiting. Among married women, there were no major differences in unmet need for contraception regarding urban-rural residence or schooling, but there were differences between those with no health insurance and those who have it, with the former group having almost doubled the level of unmet need for contraception than the latter. The number of children was also significant with those without children having a greater need for contraception for spacing compared to those with three or more children. Among sexually active women never in union, those who resided in rural areas, those who had lower levels of education and those with no children had the greatest unmet need for contraception for spacing. Table 2 shows the results of the first logistic regression model with variables associated with unmet need for spacing. After controlling for age, marital status, urban-rural residence, schooling, region of residence and access to health service, the likelihood of having unmet need for spacing increased for younger women compared to older women (OR 6.8; CI: 2.95–15.48), never in union women compared with those currently in union (OR 1.6, CI: 1.40–1.79), those with lower schooling than those with higher schooling (OR 1.4, CI: 1.26–1.56); those living in region 4 (OR 1.9; CI: 1.52–2.49) compared to region 1. Women were less likely to have unmet need for contraception for spacing if they had full access public health service (OR 0.8, CI 0.66–0.88) than those who did not. Table 3 shows the results of the second logistic regression model with the variables associated with unmet need for limiting. After controlling for age, marital status, urban residence, schooling, region of residence and access to health services, women were more likely to have unmet need for limiting if they were younger (OR 6.3; CI: 4.73–8.27), never in union (OR 3.0, CI: 2.47–3.54), lived in a rural area (OR 1.2, CI: 1.07–1.32), have less schooling (OR 1.13; CI: 1.02–1.26) and resided in region 4 (OR: 1.5; CI: 1.23–1.93) compared to women who use contraceptive methods to limit their fertility. Women were less likely to have unmet needs for limiting if they had some type of access to health service (private OR: 0.5; CI: 0.37–0.77; full public OR: 0.7; CI: 0.57–0.75; partial public OR: 0.83; CI: 0.72–0.95).

**Table 1:** Proportion of women with met and unmet need for spacing and limiting, by marital status, Mexico 2014

	Currently in union					Never in union sexually active (prev. 3 months)				
	Use for limiting	Use for spacing	Unmet need for limiting	Unmet need for spacing	Total	Use for limiting	Use for spacing	Unmet need for limiting	Unmet need for spacing	Total
	%	%	%	%	*n*	%	%	%	%	*n*
Total^a^	57.4	15.2	6.4	5.1	51,758	19.8	43.8	8.0	20.9	5039
Age group										
15–19	11.7	39.8	6.2	21.9	2307	7.6	46.9	6.7	35.8	921
20–24	23.9	37.7	6.4	15.4	6065	11.0	55.2	4.6	25.3	1634
25–29	40.0	27.7	6.5	8.6	7958	16.0	54.2	5.9	17.2	1095
30–34	58.2	16.0	6.7	3.7	8897	29.0	32.6	9.1	16.3	613
35–39	71.1	7.3	6.9	1.6	9655	41.1	19.8	18.1	5.5	343
40–44	76.9	2.6	6.5	0.4	9118	51.8	3.2	21.3	1.6	253
45–49	74.0	1.0	5.1	0.1	7758	68.3	1.7	15.6	0.6	180
Type of residence										
Rural	50.5	16.6	7.2	6.9	12,568	15.8	34.4	11.2	29.0	393
Urban	59.6	14.7	6.1	4.5	39,190	20.1	44.6	7.7	20.3	4646
Education										
< 10 years	59.8	12.3	7.1	4.9	33,676	29.4	26.2	12.2	20.4	1356
10+ years	52.9	20.6	5.0	5.4	18,082	16.3	50.3	6.5	21.1	3683
Region of residence										
Region 1	61.7	13.9	5.8	3.7	3091	20.6	51.6	7.6	15.8	841
Region 2	58.7	16.0	5.4	4.6	16,808	17.3	40.9	8.0	25.3	1657
Region 3	57.9	15.0	6.6	4.9	26,440	21.4	44.0	7.8	19.0	2315
Region 4	48.0	14.2	8.8	8.4	5419	18.1	34.1	10.6	27.4	226
Number of live births										
0	6.1	22.8	4.0	15.7	4294	9.0	51.9	5.7	25.8	3676
1	20.8	39.2	5.3	10.6	10,058	31.7	32.4	14.7	12.4	774
2	63.8	13.7	7.3	3.8	15,784	61.8	12.1	16.8	2.9	340
3 or more	79.9	3.6	6.7	1.4	21,622	83.9	2.8	8.8	0.4	249
Access to Health Services										
No Social Security Health Service	52.9	15.6	7.7	6.6	7494	16.7	45.6	8.6	22.8	1263
Partial Access Public Health Service	54.7	16.1	7.3	6.1	23,407	25.1	34.8	9.0	22.9	1326
Full Access Public Health Service	62.3	13.9	5.0	3.5	19,654	19.4	47.2	7.2	18.8	2238
Private Health Service	58.6	15.7	2.7	2.7	1203	9.0	54.2	6.6	20.8	212

^a^Note: For the total 100% of women currently in union, women who are infecund and want to have children account for 11.9%. For the total 100% of never in union women who have been sexually active in the previous 3 months, women who are infecund and want to have children account for 7.5%

**Table 2:** Adjusted odds ratios for multivariable logistic regression of factors independently associated with unmet need for spacing among women of reproductive age Mexico 2014

	Unmet need for Spacing (*n* = 13,756)		
	OR	*p*-value	[95% Conf. Interval]
Age group			
15–19	6.76	0.000	2.95–15.48
20–24	5.17	0.000	2.27–11.79
25–29	3.96	0.001	1.74–9.05
30–34	3.48	0.003	1.52–7.97
35–39	2.87	0.015	1.22–7.72
40–44	1.97	0.141	0.80–4.87
45–49	1.00		
Marital status			
Never in union	1.58	0.000	1.40–1.79
In union	1.00		
Residence			
Rural	1.11	0.076	0.99–1.25
Urban	1.00		
Education			
< 10 years	1.40	0.000	1.26–1.56
10 + years	1.00		
Region			
Region 1	1.00		
Region 2	1.23	0.066	0.99–1.54
Region 3	1.12	0.322	0.89–1.41
Region 4	1.94	0.000	1.52–2.49
Access to Health Service			
No Social Security Health Service	1.00		
Partial Access Public Health Service	0.90	0.116	0.78–1.03
Full Access Public Health Service	0.76	0.000	0.66–0.88
Private Health Service	0.77	0.116	0.56–1.07

**Table 3:** Adjusted odds ratios for multivariable logistic regression of factors independently associated with unmet need for limiting among women of reproductive age Mexico 2014

	Unmet need for Limiting (*n* = 34,407)		
	OR	*p*-value	[95% Conf]
Age group			
15–19	6.25	0.000	4.73–8.27
20–24	3.34	0.000	2.75–4.04
25–29	2.21	0.000	1.85–2.64
30–34	1.60	0.000	1.36–1.89
35–39	1.41	0.000	1.20–1.66
40–44	1.25	0.008	1.06–1.48
45–49	1.00		
Marital Status			
Never in union	2.96	0.000	2.47–3.54
In union	1.00		
Residence			
Rural	1.19	0.002	1.07–1.32
Urban	1.00		
Education			
< 10 years	1.13	0.023	1.02–1.26
10 + years	1.00		
Region			
Region 1	1.00		
Region 2	0.93	0.472	0.75–1.14
Region 3	1.01	0.955	0.81–1.25
Region 4	1.54	0.000	1.23–1.93
Access to Health Service			
No Social Security Health Service	1.00		
Partial Access Public Health Service	0.83	0.007	0.72–0.95
Full Access Public Health Service	0.65	0.000	0.57–0.75
Private Health Service	0.54	0.001	0.37–0.77

## Discussion

Unmet need for contraception is an important health problem for women in Mexico. Our study found that the level of unmet need for contraception among married women was not very high (11.5%), and was similar to the estimated level for Latin America, reported for 2010 at 10.5% [4]. Additionally, the use of contraceptive methods was widespread among Mexican women as a whole. However, the analysis showed important differences regarding sociodemographic characteristics and demonstrated that new reproductive desires and sexual behaviours are not being accompanied by appropriate public contraceptive policies. Age was the most significant variable in both models (spacing and limiting). Young women, both currently in union and never in union, had difficulty using the contraceptive methods they required according to their reproductive desires, both for spacing and for limiting, and therefore, had a high probability of having unintended pregnancies. Recent estimates indicate that in Mexico 38 out of 1000 women of reproductive age have had clandestine abortions as a result of not having the necessary contraceptive methods [19]. Inconsistent use of contraceptives is also a problem experienced by young women [20]. The second most significant variable was marital status, where women never in union had a much greater unmet need for contraception than women currently in union. The country’s family planning services were originally designed exclusively for married women, and despite evidence of increased sexual initiation prior to marriage, the need for contraception has not been met adequately for the younger population [7]. Women living in the country’s poorest region, which is the region with the highest proportion of indigenous population (Region 4), have greater unmet needs for both spacing and limiting compared to women residing in the capital of the country. This finding clearly shows that services are insufficient in those areas of the country where women have traditionally had the highest fertility rates, but now have a greater desire to space and limit births. As previously reported [7], having health insurance reduces the unmet need for contraception both to limit and to space. One characteristic of contraceptive use by Mexican women is that they tend to be sterilized at an early age. Thus, in the age group 30–34 years, sterilization ranks highest in contraceptive methods used, and ranks second for the 25–29 years age group [7]. Since performing sterilizations require medical facilities, having medical insurance to cover the medical procedure will make a difference. The ORs of the second model (need to limit), reflect that having any type of access to health service significantly reduced unmet need for contraception (for limiting). In the Latin American region, there has been a high use of sterilization [1], and although the level varies by country, Mexico is in the group of countries that uses this method the most [21].

A limitation of the study is that the estimation of unmet need for contraception did not consider the particular efficacy of different contraceptive methods. Women who used both modern and traditional methods were defined as having met needs, even though some methods are inefficient in the prevention of pregnancy [4]. However, this limitation has little weight in the case of Mexico, as only 4.2% of the women in this analysis use traditional methods (4.2% of currently married and 4.1% of never married women who were sexually active).

## Conclusions

This research has shown the urgency of focussing on the country’s young population in terms of contraceptive services. Younger women, both currently in union and never in union, had the highest unmet needs of contraception for spacing and limiting. This points out to the need to redesign the country’s contraceptive programmes. Previous research has shown that well-organized family planning programmes have a direct and indirect impact on reducing unmet need for contraception [4, 6]. In Mexico, priority should be given to providing contraceptive services to the young population in general, as well as to women in the poorest region of the country.

## Abbreviations

CONEVAL: National Council for the Evaluation of Social Development Policy
DHS: Demographic and Health Surveys
ENADID: National Survey of Demographic Dynamics
INEGI: National Institute of Statistics and Geography
